# Enhanced Toxicity of Polymethylmethacrylate Microparticles on Cells and Tissue of the Marine Mussel *Mytilus trossulus* After UV Irradiation

**DOI:** 10.3390/toxics13100818

**Published:** 2025-09-26

**Authors:** Nadezhda Vladimirovna Dovzhenko, Victor Pavlovich Chelomin, Sergey Petrovich Kukla, Valentina Vladimirovna Slobodskova, Andrey Alexandrovich Mazur

**Affiliations:** V.I. Il’ichev Pacific Oceanological Institute, Far Eastern Branch, Russian Academy of Sciences, Vladivostok 690041, Russia

**Keywords:** artificial aging, biomarkers, microplastic, mussel, polymethylmethacrylate, ultraviolet irradiation

## Abstract

In the marine environment, plastic fragments are constantly engaged in a complex degradation process under exposure to various physical and chemical factors, one of which is ultraviolet (UV) radiation. These processes result in the formation of smaller micro- and nano-sized plastic particles, which are highly bioavailable to marine organisms. To clarify the toxicological effects of the exposure of degraded plastic on the marine organisms, the model used in this study was the Pacific mussel *Mytilus trossulus* and polymethylmethacrylate (PMMA), which is commonly found in marine debris. Using molecular and biochemical markers (DNA damage, lysosomal membrane stability, integral antiradical activity (IAA) of biological samples, and malondialdehyde (MDA) as a product of lipid peroxidation), the toxicity of pristine PMMA and photoaged (PMMA-UV) particles was assessed. Using Fourier transform infrared spectroscopy, the characteristics of the macromolecular changes in the chemical structure of PMMA-UV were obtained, with an oxidation index of 6.83 ± 0.46, compared to the pristine PMMA of 5.15 ± 0.54. Using a laser analyzer, the sizes of PMMA particles were determined, and it was found that after UV irradiation, the ratio of size groups changed—the proportion of particles with sizes of 500–1000 μm decreased, and the number of particles with sizes of 50–125 μm increased twofold. Analysis of mussel cell viability showed that after exposure to both types of PMMA microparticles, there was a decrease in the ability to retain neutral red dye in lysosomes: PMMA and PMMA-UV had a similar effect on hemocytes, reducing dye retention in cells to 55.2 ± 3.24% and 61.1 ± 1.99%, respectively. In gill and digestive gland cells, PMMA-UV particles reduced the stability of lysosomal membranes to a greater extent than PMMA. After PMMA and PMMA-UV particle exposure, the levels of DNA damage were as follows: in hemocytes, 10.1 ± 1.4% and 12.7 ± 0.8%, respectively; in gills, 7.8 ± 1.1% and 14.4 ± 2.9%, respectively; and in the digestive gland, 19.0 ± 1.3% and 21.9 ± 2.8%, respectively, according to the control values 3.6 ± 1.3%, 4.6 ± 1.1%, 5.1 ± 1.5%, respectively. According to the results of biochemical markers, the reaction of mussels to the presence of PMMA and PMMA-UV particles in the environment was tissue-specific: in the cells of the digestive gland, the level of IAA increased by 2 and 1.3 times compared to the control group of mussels (76.22 ± 6.77 nmol trolox/g wet weight and 52.43 ± 2.36 nmol trolox/g wet, respectively), while in the gill cells, the non-significant increase in antiradical activity was noted. An increase in MDA content was also observed in gill cells (255.8 ± 9.12 nmol MDA/g wet weight and 263.46 ± 9.45 nmol MDA/g wet weight, respectively) compared with the control group. This study showed that UV irradiation of PMMA microparticles increases their bioavailability and toxicity to *M. trossulus*.

## 1. Introduction

The massive entry of plastic waste and its accumulation along the coastlines of marginal seas, especially in littoral zones, river mouths, lagoons, and bays with restricted water exchange [1,2,3], have unpredictable environmental effects, the identification and assessment of which require in-depth ecotoxicological studies. Specialists’ concerns are related not only to the concentration of plastic waste in these areas, but also to the transformation of different types of polymers in this environment—plastic weathering or aging. A wide range of mechanical (abrasion by sand and stones, wave and wind mixing), physicochemical (sunlight, oxygen, temperature changes, dissolved salts and metals with variable valence) and biological (microorganisms, fouling organisms) factors are involved in the aging of plastic. As a result of these processes, a cascade of physical and chemical transformations is initiated in the structure of the polymer chains, and the plastic becomes brittle and gradually breaks into particles of various sizes, from micro- to nanoplastics [4,5]. These processes not only lead to the formation of plastic micro- and nanoparticles, significantly increasing their bioavailability for a large number of aquatic organisms, but are also accompanied by significant changes in the physicochemical properties of the polymers themselves [6,7,8,9,10,11,12]. In addition, aging plastic releases various chemical decomposition and incomplete polymerization products (monomers and oligomers) into the marine environment, as well as a wide range of chemical additives, which are added to polymers during synthesis to give them certain physical properties, among which the most widely used are relatively stable and highly toxic chemical compounds (bisphenol A, phthalates, polychlorinated biphenyls, metals, etc.) [13,14,15]. Therefore, the accumulation of huge masses of plastic in the coastal zone can reasonably be considered a powerful source of micro- and nanoplastics and a complex mixture of chemicals, which poses a serious potential threat to various marine organisms and, above all, to littoral inhabitants [1,2,16,17,18].

These concerns are confirmed by experimental studies showing that different types of plastic microparticles can initiate disorders in marine organisms, manifesting at different levels of organization, including the molecular level [12,18,19,20,21,22,23,24,25,26,27,28,29,30]. Among them, there is a growing number of publications indicating an increase in polymer toxicity following oxidative degradation, i.e., aging [10,31,32,33,34,35]. However, analysis of these literature sources showed that most of the laboratory studies assessing the toxic risks of microplastics interacting with marine organisms were conducted using a limited number of plastic types, such as polystyrene (PS), polyethylene (PE), and polypropylene (PP), each of which has its own characteristics—monomers, specific additives, and physicochemical properties. There is insufficient research characterizing the interaction of marine organisms with other types of polymers, which are not only widely present in the environment but are also synthesized from more toxic monomers.

Thus, polymethylmethacrylate (PMMA), along with PS, PE and PP, is also considered widespread in the environment and is often found in marine debris due to its widespread use in human economic and domestic activities (optical lenses, touch screens, appliances, paint resins, abrasives, cosmetics, prosthetic materials, etc.) [35,36,37,38,39,40]. The impact of PMMA microparticles on living organisms deserves special attention because, unlike PS, PE, and PP, this polymer is a product of the polymerization of acrylic acid derivatives, which are toxic, and PMMA itself, according to current classifications, is classified as a potentially hazardous polymer [41,42].

However, despite the interest in this polymer, studies on the toxicological characteristics of PMMA are limited. Thus, it has been established that PMMA nanoparticles cause disturbances in the gene expression associated with lipid metabolism in the sea bass *Dicentrarchus labrax* [19]. In the presence of PMMA microparticles in the external environment, the successful fertilization rate of eggs decreased and various cytogenetic damages appeared in the embryos and larvae of the sea urchins *Paracentrotus lividus* and *Sphaerechinus granularis* [43,44]. In the benthic polychaete *Hediste diversicolor*, PMMA microparticles affected the antioxidant system and energy metabolism [40]. Also, it was shown that PMMA microparticles, entering the digestive system of the benthic gastropod *Littorina brevicula* with food, caused DNA damage in the digestive gland cells [18].

In this study, considering that the feeding habits of marine organisms are important in their susceptibility to microplastics, to determine the pathways of various toxicants into the body, the benthic littoral mollusk *Mytilus trossulus* was used as a model organism. Mussels, as filter feeders, are exposed to additional risks because, by filtering huge amounts of seawater through their gills, they extract micro- and nanoplastics, which can penetrate the circulating hemolymph and then concentrate in the organs of the digestive system [45]. In this regard, the issue of the negative consequences for cells that come into contact with the microparticles of hazardous polymers such as PMMA is important but has not been sufficiently studied, which explains the relevance of this study. In addition, PMMA, like all types of plastic, is found in the environment in various degrees of aging, so there is a need for additional research on the role of aging in the development of negative processes. Thus, to identify and assess the real toxic potential of PMMA in this study, particles of this polymer were exposed to UV irradiation for 120 h. Currently, this approach is widely used in laboratory studies to develop and accelerate the aging processes of various types of polymers [8,9,10,46,47].

In this context, in order to clarify the toxicological effects of partially aged plastic, comparative studies of the cyto- and genotoxic potential of pristine and photoaging PMMA microparticles in the mussel *M. trossulus* were conducted.

## 2. Materials and Methods

### 2.1. PMMA and PMMA-UV Preparation and Characterization

In the study, we used polymethylmethacrylate (PMMA) with the trade name “Protacryl M” (Nikadent LLC., St. Petersburg, Russia). The polymer was in the form of a dry powder. Two types of granules were used in the experiment: the pristine PMMA (PMMA) and UV-irradiated (aged) PMMA (PMMA-UV). The polymer photodegradation process was simulated in laboratory conditions: PMMA granules were continuously irradiated in a Petri dish for 120 h using a Supratec HTC 400-241 lamp (Osram, Munich, Germany, 240 W, a frequency of 50 Hz and wavelength 275–470 nm). The distance from the lamp to the surface of the plastic particles was 3 cm.

The identification of PMMA and PMMA-UV was performed using an IRAffinity-1S IR Fourier transform spectrophotometer (Shimadzu, Kyoto, Japan) with a total internal reflection attachment. The spectra were recorded as 32 scans in the spectral range of 4000–400 cm^−1^, with a spectral resolution of 4 cm^−1^. The background was measured using the same settings against air. The obtained spectra were processed using LabSolutions IR 2.27 software (Shimadzu, Kyoto, Japan). The content of the functional groups in all PMMA samples was calculated using the oxidation index (IO) [11].

The particle sizes of the pristine PMMA and PMMA-UV were determined using an Analysette 22 NanoTec laser diffraction particle size analyzer (Fritsch, Idar-Oberstein, Germany). The dispersion medium was created using a certified PAV-901 standard (Fritsch, Idar-Oberstein, Germany). A certified F-500 standard (Fritsch, Idar-Oberstein, Germany) was used for calibration. The obtained particle size spectra are presented as a percentage of volume on a logarithmic scale, the values of which were calculated based on the particle diameter. Statistical parameters were calculated using the GRADISTAT v.8.0 software package [48].

### 2.2. Experiment Design

*M. trossulus* mussels were collected in July 2023 in Alekseev Bay (Amur Bay, Sea of Japan) at the experimental site of the Popov Island Marine Experimental Station of the V.I. Il’ichev Pacific Oceanological Institute of the Far Eastern Branch of the Russian Academy of Sciences. Adult individuals of one generation (measuring size = 5.06 ± 0.4 cm) were used in the experiment. Seawater was taken into aquariums from Alekseev Bay. Under laboratory conditions, the mussels were cleaned of fouling organisms and then placed in a glass aquarium with a water volume of 5 L per mussel. The mollusks were acclimated under constant conditions: water temperature +18 ± 0.3 °C, salinity 32.6 ± 0.30‰, pH 8.2 ± 0.2, O_2_ 7.5 ± 0.3 mg/L and photoperiod 16 h/8 h (light/dark). The acclimation period lasted 168 h. The seawater was replaced twice a day. The hydrochemical parameters of the water and the temperature were recorded every 12 h using a portable device, Combo Water Quality Meter IP67, mod. 86031 (AZ, Taichung, China). In the experiment, seawater filtered through a three-fraction gravel filter and treated with ultraviolet light was used.

After acclimation, the mussels were divided into three experimental groups. The first group consisted of mussels without exposure to microplastics (control), the second group consisted of mussels exposed to the pristine PMMA, and the third group consisted of mussels exposed to PMMA-UV. Each group had 3 replicates.

Each group was a glass tank containing 9 mussels, with 0.5 L of water per mussel. To eliminate the presence of pathogens and suspended solids, including microplastics, the seawater used in the experiment was filtered and sterilized. The concentrations of PMMA and PMMA-UV in the water were 20 mg/L, respectively. The weights of PMMA and PMMA-UV were measured on Shimadzu AW 220 analytical scales (Shimadzu, Kyoto, Japan). The water in the tanks was aerated using compressors, which actively mixed the water and prevented microparticles from settling to the bottom. The experiment lasted 72 h under the following constant conditions: water temperature +18 ± 0.3 °C, salinity 32.6 ± 0.30‰, pH 8.2 ± 0.2, O_2_ 7.5 ± 0.3 mg/L and photoperiod 16 h/8 h (light/dark). The hydrochemical parameters of the water and temperature were recorded every 12 h. Throughout the experimental period, the clear and PMMA/PMMA-UV-containing water types were changed every 24 h. During the experiment, the filtration system was turned off, and the mussels were not fed additionally.

No mollusk mortality was observed during the exposure. At the end of the experiment, 27 *M. trossulus* individuals from each control and experimental group were used to determine the cytochemical and biochemical parameters. The mussels were taken out of the tank one at a time, and the shell was dried with filter paper and placed on a surface cooled to 4 °C. The shell of the mollusks was opened with a medical scalpel. The gills and digestive gland were separated from the mollusk using scissors and tweezers. All procedures in this study and methods of mussel disposal after the experiment were approved by the Bioethics Commission of the V.I. Il’ichev Pacific Oceanological Institute of the Far Eastern Branch of the Russian Academy of Sciences (protocol No. 16, approved on 15 April 2021), Vladivostok, Russia.

### 2.3. Cytochemical Assays

The cytotoxicity of the tested PMMA and PMMA-UV was determined using the neutral red (NR) dye method [49,50]. The analysis was based on the absorption and accumulation of dye in the lysosomes of living cells. Provided that the cell is viable, NR is absorbed and retained inside to the maximum extent, whereas when cells are damaged, the rate of NR accumulation decreases, and when cells die, the dye is not retained [50].

The hemolymph, gills, and digestive glands were used for analysis. Hemolymph was collected using a syringe from the adductor muscle. A 100 μL amount of hemolymph was collected from each mussel, combining hemolymph from three mollusks into one sample. Pieces of tissue from the gills and digestive gland of the dissected mussels were transferred to glass buckets with phosphate buffer (0.05 M, pH 7.4) and carefully crushed with scissors. Then, to remove large particles, the cell suspension was filtered through a mesh filter with a mesh size of 100 μm.

A 300 μL amount of hemolymph and 300 μL of a suspension of gill and digestive gland cells were collected. Then, 60 μL of NR solution with a concentration of 50 μg/mL was added to 300 μL of the cell suspension. The resulting mixture was incubated on a TS-100C thermoshaker (Biosan, Riga, Latvia) at 37 °C for 60 min. After incubation, the cells were washed twice to remove dye residues with phosphate buffer (0.05 M, pH 7.4) in a volume of 300 μL. Then, 300 μL of acetic acid solution in ethanol (ratio 1% acetic acid/50% ethanol) was added to an Eppendorf-type microtube with the washed cells and incubated at 20 °C for 15 min. The optical density of the samples was measured on a UV-2550 spectrophotometer (Shimadzu, Kyoto, Japan) at 540 nm. There were three repetitions for each study: control/PMMA/PMMA-UV. The calculation of NR cell retention was performed based on the estimated number of cells in each sample tested (hemolymph 3,900,000 cells, gills 6,500,000 cells, digestive glands 54,000,000 cells). To determine the working concentrations of cells, counts were performed in a Goryaev chamber. Counts were performed in triplicate for each experimental group cell, and viability was expressed as the percentage compared to the control [49].

To quantitatively assess the genotoxicity of the polymethylmethacrylate particles, an alkaline version of the comet assay, adapted for marine organisms, was used [34]. To prepare the cell suspension, the gills and digestive glands of mussels were dissected on ice. The tissue was then quickly transferred to a glass beaker containing isotonic solution and carefully chopped with scissors. The hemolymph suspension was used without preparation. Gel slides were prepared in two stages: first, 50 μL of cell suspension was mixed with 100 μL of 1% low-melting-point agarose (MP Biomedicals, Eschwege, Germany) and applied to a slide precoated with 1% agarose with a normal melting point and covered with a cover slip. After the gel had solidified, the slides were transferred to a lysis solution (2.5 M NaCl; 0.1 M EDTA-Na_2_; 1% Triton X-100; 10% DMSO; 0.02 M Tris, pH 10) and incubated in the dark at 4 °C for 1 h. The slides were then washed with cold (4 °C) distilled water and kept in 4 °C electrophoresis solution (300 mM NaOH, 1 mM EDTA-Na_2_, pH > 13) in the dark for 40 min, and then electrophoresis was started for 20 min. After electrophoresis, the slides were immediately neutralized with Tris-HCl (0.4 M, pH 7.4) and washed with distilled water. After drying, the slides were stained with SYBR Green I fluorescent dye. DNA comet images were captured using an AxioImager A1 scanning fluorescence microscope (Carl Zeiss, Oberkochen, Germany) with an AxioCam MRc digital camera. Casp 1.2.2 software (CASPlab, Wrocław, Poland) was used to process the digital images. DNA comets obtained for mussels from the control and experimental groups were analyzed for each mollusk (1 slide = 1 mussel). Each slide contained at least 100 comets. The percentage of DNA damage (% DNA in the tail) was determined for each comet.

### 2.4. Biochemical Assays

To determine the integral antiradical activity (IAA) and malondialdehyde (MDA) content, pieces of gills and digestive gland were taken, weighed on a scale, and then homogenized on a magnetic homogenizer Silent Crusher S (Heidolph Instruments, Schwabach, Germany) at a temperature of +4 ° C in phosphate buffer (0.1 M, pH 7.0) in a ratio of 1:10 g of raw tissue/mL. The resulting homogenates were centrifuged at 10,000 rpm at a temperature of +4 °C for 40 min.

The IAA in tissue homogenates was determined using a method based on the ability of the antioxidant system of cells to suppress the oxidation reaction of 2,2′-azobis (3-ethylbenzothiazoline-6-sulfonate) (ABTS) by peroxyl and alkoxyl radicals formed during the thermal decomposition of 2,2′-azobis (2-methyl-aminopropane) dihydrochloride (AAPH) [51]. For analysis, 2.6 mL of phosphate buffer (0.1 M, pH 7.0), 10 μL of homogenate, and ABTS solution were added to a quartz cuvette. The reaction mixture was incubated at 37 °C for 5 min. Then, maintaining a temperature of 37 °C, 300 μL of AAPH solution was added to the sample and its optical density was measured in kinetics on a UV-2550 spectrophotometer (Shimadzu, Kyoto, Japan) at a wavelength of 414 nm. The IAA value was calculated using a calibration curve constructed for 6-hydroxy-2,5,7,8-tetramethylchloraman-2-carboxylic acid (trolox) (Sigma Aldrich, St. Louis, MI, USA).

The concentration of malondialdehyde (MDA) as a product of lipid peroxidation in the homogenates was determined using a colorimetric method [52]. To 0.75 mL of the homogenate, 0.5 mL of 30% trichloroacetic acid (TCA) and 0.5 mL of 0.75% 2-thiobarbituric acid (TBA) were added sequentially. To initiate the reaction of lipid peroxides with TBA, the mixture was thoroughly mixed and heated in a water bath to 95 °C for 20 min. To stop the reaction after thermal incubation, the samples were rapidly cooled in ice water and centrifuged for 30 min at 3000 rpm. The optical density of the samples was measured on a UV-2550 spectrophotometer (Shimadzu, Kyoto, Japan) at wavelengths of 580 nm and 532 nm. The molar extinction coefficient (E1M = 1.56 × 10^5^) was used to calculate the MDA concentration, which was expressed in nmol/g wet weight.

### 2.5. Statistical Processing

Statistical processing of the obtained results was performed using MS Office Excel and Statistica 10 software packages (StatSoft, Tulsa, OK, USA). The reliability of the differences between the samples was determined using the nonparametric Mann–Whitney criterion. Differences were considered statistically significant at *p* < 0.05.

## 3. Results

### 3.1. The Effect of UV on the Composition and Size of PMMA Microparticles

**Figure 1:**
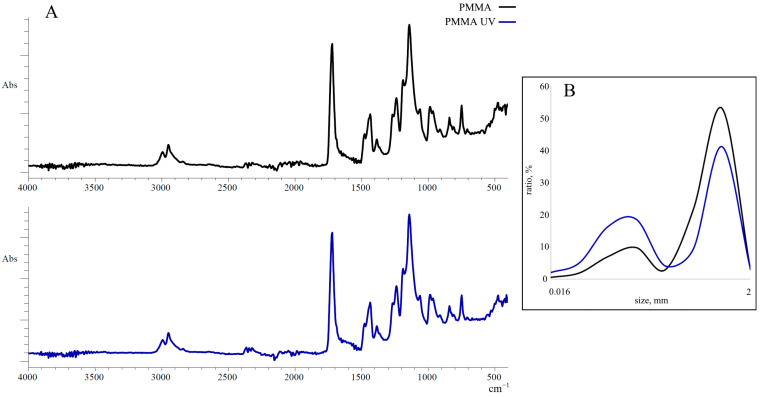
(**A**) FTIR spectra of pristine polymethylmethacrylate (PMMA) and ultraviolet-irradiated polymethylmethacrylate (PMMA-UV); (**B**) PMMA and PMMA-UV particle size distribution.

The PMMA used in our work is an acrylic polymer of the low-temperature polymerization of methyl methacrylate and a fluorine-containing copolymer. Samples of this polymer were characterized using Fourier transform infrared spectroscopy (FTIR) to identify changes in their chemical structure caused by UV irradiation. According to the FTIR data shown in Figure 1A, the PMMA spectra contain spectral bands typical of this polymer.

The strong bands observed at 1723 cm^−1^ and 1144 cm^−1^ are vibrations of the carbonyl (R=O) and carboxyl (R–O) groups, respectively. The spectral bands in the range 2900–2950 cm^−1^ and 750 cm^−1^ correspond to the stretching of C–H bonds in CH_3_ and CH_2_ groups, while the bands at 1435 cm^−1^ and 1386 cm^−1^ are bending (deformation) vibrations of the same groups. The stretching of C–C bonds is indicated by the line at 986 cm^−1^.

A similar pattern was observed in PMMA-UV samples. After baseline correction and comparison of the reference (unchanged) peak at 750 cm^−1^ relative to the line at 1723 cm^−1^, the oxidation index (OI) was calculated. The calculations showed that after UV irradiation under our experimental conditions, the OI in the PMMA samples increased by almost 25% (5.15 ± 0.54 and 6.83 ± 0.46, respectively), which indicates changes in the macromolecular structure of the polymer.

According to the laser analyzer data, we used PMMA samples in our experiments, with particle sizes ranging from a few micrometers to 2 mm (Figure 1B). Particles ranging from 50 μm to 1 mm accounted for more than 90% of all particles. In the quantitative distribution of particles by size, two pools are distinguished. The first contains polymer particles with a size of 50–125 μm, and the second contains particles with a size of 500–1000 μm. After the UV irradiation of PMMA, the bimodal nature of the distribution of microparticles was preserved. However, analysis of these data shows that after irradiation, the proportion of particles with sizes of 500–1000 μm decreased, while the content of microparticles of 50–125 μm increased significantly.

### 3.2. Cell Viability Analysis

**Figure 2:**
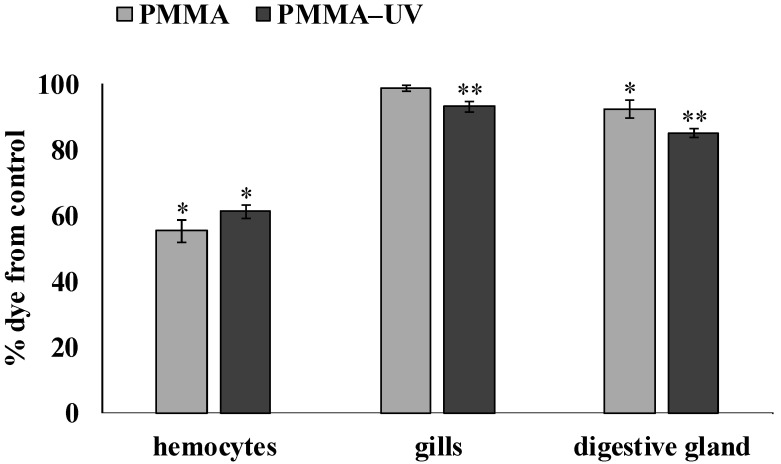
Results of NR test after pristine polymethylmethacrylate (PMMA) and ultraviolet-irradiated polymethylmethacrylate (PMMA-UV) exposure (mean ± sd); *—difference from control; **—indicates significant differences compared to control and exposure to pristine PMMA (*p* < 0.05).

The viability of hemocytes, gill and digestive gland cells of mussels was analyzed using neutral red (NR) dye. The results of this cytochemical test are presented in Figure 2.

In experimental mollusks, after exposure to both types of polymer microparticles (PMMA or PMMA-UV), a significant decrease in the ability to retain dye in lysosomes was observed relative to the control. In hemocytes, this indicator decreased to 55.2 ± 3.24% (*p* = 0.017) and 61.1 ± 1.99% (*p* = 0.021), respectively, and in digestive gland cells to 92.3 ± 2.6% (*p* = 0.040) and 85 ± 1.42% (*p* = 0.036), respectively. In the gills, a significant decrease was observed only after exposure to PMMA-UV (93 ± 1.6% (*p* = 0.041)). It is characteristic that the effect of PMMA-UV had a significantly stronger effect on the ability to retain dye in lysosomes compared to the effect of the pristine PMMA in the gills (*p* = 0.044) and digestive gland (*p* = 0.043).

### 3.3. Pro-Oxidative Processes Indicators

**Figure 3:**
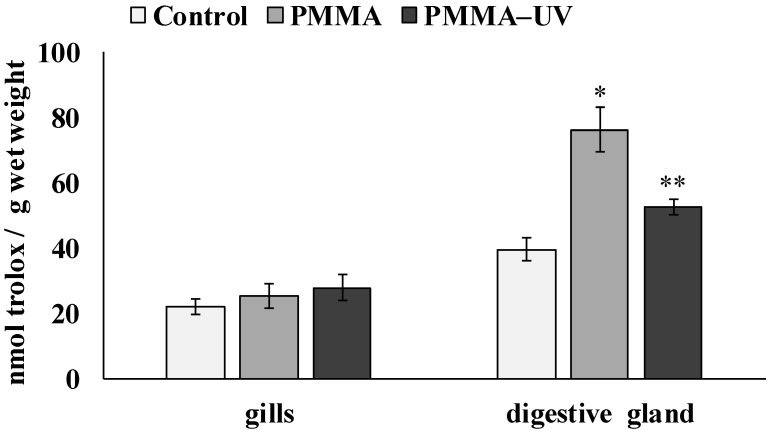
IAA in *M. trossulus* tissues after pristine polymethylmethacrylate (PMMA) and ultraviolet-irradiated polymethylmethacrylate (PMMA-UV) exposure (mean ± sd); *—indicates significant differences compared to the control; **—indicates significant differences compared to control and exposure to pristine PMMA (*p* < 0.05).

The effect of the microparticles of both types of PMMA on the development of oxidative stress processes in the cells of the digestive gland and gills of mollusks was assessed by analyzing the integral ability of the low-molecular-weight link of the antioxidative system of mussels to neutralize the peroxyl radical (ROO·). Figure 3 shows the values of IAA measured in the tissues of the control group of mussels and the experimental groups of mussels after exposure to PMMA and PMMA-UV microparticles, respectively.

According to these results, the reaction of experimental mussels to the presence of both types of microplastics in the environment was tissue-specific. While a slight increase in antiradical activity was observed in the gill cells (control—22.09 ± 2.36 nmol trolox/g wet weight; PMMA—25.4 ± 3.75 nmol trolox/g wet weight; PMMA-UV—27.82 ± 4.08 nmol trolox/g wet weight), the level of antiradical activity in the digestive gland cells increased significantly compared to the control group of mussels by 2 (*p* = 0.011) and 1.3 (*p* = 0.018) times, respectively (control—39.55 ± 3.69 nmol trolox/g wet weight; PMMA—76.22 ± 6.77 nmol trolox/g wet weight; PMMA-UV—52.43 ± 2.36 nmol trolox/g wet weight). The integral response of digestive gland cells differed significantly between the two experimental groups (*p* = 0.024).

**Figure 4:**
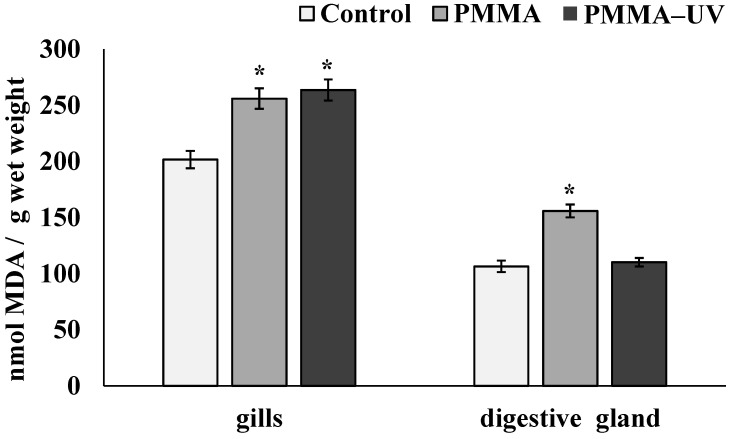
MDA concentration in *M. trossulus* tissues after pristine polymethylmethacrylate (PMMA) and ultraviolet-irradiated polymethylmethacrylate (PMMA-UV) exposure (mean ± sd); *—difference from control (*p* < 0.05).

Figure 4 shows the levels of MDA in the tissues of control and experimental mussels. In mollusks exposed to both PMMA microparticles, the content of these products in the cells of the gills significantly increased compared to the control group of mollusks (control—201.52 ± 7.69 nmol/g wet weight; PMMA—255.8 ± 9.12 nmol/g wet weight (*p* = 0.028); PMMA-UV—263.44 ± 9.45 nmol/g wet weight (*p* = 0.027). In the digestive gland, this indicator increased only after pristine PMMA exposure (control—106.4 ± 5.15 nmol/g wet weight; PMMA—155.77 ± 5.78 nmol/g wet weight (*p* = 0.015); PMMA-UV—110.04 ± 3.76 nmol/g wet weight).

### 3.4. Genotoxicity

**Figure 5:**
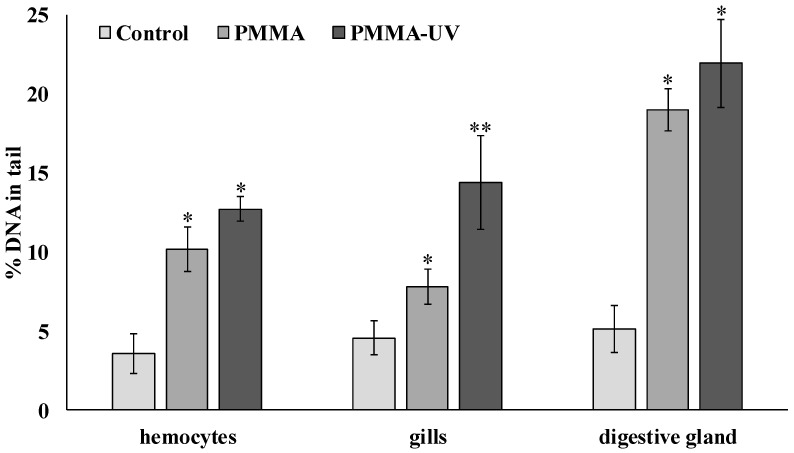
Comet assay results after pristine polymethylmethacrylate (PMMA) and ultraviolet-irradiated polymethylmethacrylate (PMMA-UV) exposure (mean ± sd). *—difference from control (*p* < 0.05). **—indicates significant differences compared to control and exposure to pristine PMMA.

DNA damage level was assessed in the individual mussel cells as a result of exposure to PMMA microparticles using the DNA assay. Figure 5 shows the results characterizing the average level of damage to the nuclear DNA molecule (% of DNA migrating in the electric field to the “tail” of the comet) in the cells of the control and experimental mussels.

The results show that both types of PMMA microparticles induce increased levels of DNA damage in all experimental mollusk cells studied, compared to control. At the same time, after exposure of mussels to pristine PMMA microparticles, the average level of DNA damage increased significantly and amounted to 19.0 ± 1.3% (*p* = 0.008) in digestive gland cells, 10.1 ± 1.4% (*p* = 0.011) in hemocytes, and 7.8 ± 1.1% (*p* = 0.039) in gill cells, which are several times higher than the values in the corresponding cells of the control mussels.

PMMA-UV exposure showed increased DNA damage compared to PMMA. In digestive gland cells and hemocytes, artificially aged polymer microparticles slightly increased the genotoxic effect to 21.9 ± 2.8% and 12.7 ± 0.8%, respectively. However, in gill cells, PMMA-UV microparticles significantly increased nuclear DNA fragmentation compared to pristine microparticles, reaching values of 14.4 ± 2.9% (*p* = 0.041).

**Figure 6:**
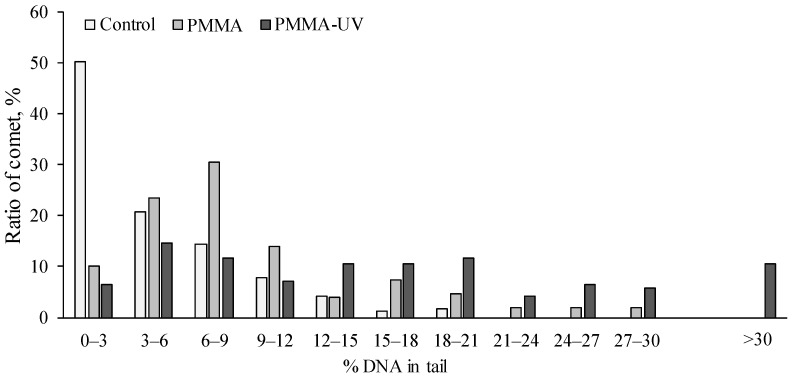
The distribution of *M. trossulus* gill cells of control and experimental groups (pristine polymethylmethacrylate (PMMA) and ultraviolet-irradiated polymethylmethacrylate (PMMA-UV)) according to DNA damage.

Therefore, to more clearly identify the differences in the impact of both types of microplastics on the cellular genome, we grouped the entire array of comets obtained in the experiments according to the level of DNA fragmentation using gill cells as an example. The frequency of occurrence of these comet groups, corresponding to the level of DNA damage, is shown in Figure 6. Analysis of the data presented in the diagrams shows that in mussel gills after exposure to microparticles of both types of PMMA, cells with a level of DNA damage more than 20% were recorded to be absent in the gills of control mollusks.

It should be noted that in the gills of mussels exposed to pristine PMMA microparticles, such cells account for no more than 5–6%, whereas in the gills of experimental mollusks after exposure to PMMA-UV, the content of these cells increases to 27%. A similar trend is observed in experimental mollusks in hemocytes and digestive gland cells. 

## 4. Discussion

As is well known, PMMA is a heat-resistant low-density polymer, and its synthesis involves the use of monomers and chemical compounds that are classified in some classifications as potentially highly toxic [41,43]. It is noteworthy that, at present, copolymers of acrylic acid derivatives, such as acrylonitrile butadiene styrene and styrene acrylonitrile, are found in the tissues of commercial bivalve species [53]. The potential danger of this polymer has been clearly demonstrated in isolated studies involving marine organisms of various trophic levels with different feeding habits. It has been established that exposure to PMMA with the planktonic microalgae *Rhodomonas baltica* and *Thalassiosira pseudonana* led to a number of physiological (decreased viability, photosynthesis, membrane integrity), biochemical (metabolic restructuring, increased reactive oxygen species (ROS) formation), and ultrastructural changes [37,54]. In the benthic marine polychaete *Hediste diversicolor*, the effect of this polymer manifested itself in behavioral changes, a decrease in the regeneration rate and energy metabolism, and an increase in the antioxidant enzyme activity and protein carbonyl content [40,55]. Experimental feeding of the benthic gnawing gastropod *Littorina brevicula* with a food substrate containing PMMA microparticles stimulated the development of oxidative stress processes in the cells of the digestive system [18]. Analysis of the results of acute and chronic experiments involving marine filter-feeding mollusks (*Crassostrea gigas* oysters and *Mytilus galloprovincialis* and *Semimytilus algosus*) showed that PMMA polymer particles overcome tissue barriers and penetrate the gills, digestive gland, and hemocytes, reducing viability and the formation of byssus threads [56,57,58]. In addition, in the marine fish *Sparus aurata*, particles of this polymer induced serious damage at the cellular and molecular levels [59]. In the embryos and larvae of zebrafish (*Danio rerio*), PMMA increased mortality and peroxide damage [60]. An example of the toxicity of PMMA particles is provided by the study by Zheng et al. (2025), which showed that in laboratory mice, this polymer accumulated in the liver and colon and caused lipid metabolism disorders [61].

The present results of experiments using *Mytilus trossulus* confirm the biological activity of the PMMA microparticles. This was clearly demonstrated by changes in biochemical marker indicators (NR, IAA, MDA, and DNA damage) after exposure of mussels to PMMA. Note that the reaction of these biochemical indicators to the impact of microplastics is tissue-specific. This may be caused by the different sensitivity of different types of cells/organs to plastic microparticles. In addition, the tissue-specific reaction of markers may, to a certain extent, be determined by the varying load on individual organs, which depends on the method of absorption (penetration or nutrition) and the nature of the distribution of microplastics between the organs and tissues of the body. It is known that in mussels, as a typical representative of filter-feeding mollusks, small (<96 μm) microplastic particles penetrate the body through the gills [45,62,63]. Larger particles are retained by mucus, forming agglomerates that are either removed as pseudofeces or transported to the oral cavity by the movement of gill cilia and enter the digestive system. Furthermore, depending on their size, microplastic particles penetrate the hemolymph and hemocytes and are distributed in individual tissues, accumulating mainly in the digestive gland [57,64]. This is confirmed by large-scale studies of various species of mollusks, presented in a review by Silva and colleagues [53], which shows that the tissues of commercial mollusks contain polymer microparticles of a wide range of sizes, but generally not exceeding 1000 μm. There is also experimental data showing that in the hemolymph, gills, and intestines of bivalve mollusks, the most common size of polymer microparticles is 10 μm, and the maximum particle size for particle retention in *M. edulis* (a species similar in size and physiology to *M. trossulus*) is 10–30 μm [45,65].

The above considerations give reason to believe that the sizes of a significant portion (90%) of the PMMA microparticles used in our work are within the range of bioavailability for absorption by mussels. Although specific studies of the distribution of PMMA microparticles of different sizes in mussel tissues/organs are beyond the scope of this work, the results agree with the opinion of a number of authors [45,57,66,67,68] that it is the cells of the gills, digestive gland, and hemocytes that experience varying degrees of stress when mussels absorb plastic microparticles. For example, in a study of the impact of PMMA particles measuring 10 and 50 μm on *M. galloprovincialis*, it was found that the retention and redistribution of microplastics in the gills and digestive gland of mussels depended on the particle size and exposure time. Particles measuring 10 μm were found in histological sections of the gills and digestive gland tubules, while particles of all sizes were found in the hemolymph. The authors also demonstrated the tissue specificity of microplastic accumulation—for example, in the ducts of the digestive gland, the occurrence of particles measuring 10 μm was 25 pieces per individual, while in the gills it was 4–5 times less: 5–8 pieces per individual, regardless of the concentration of microparticles and the duration of the experiment [57].

The results of cell marker evaluation in these tissues showed that PMMA microparticles have an active effect on metabolic processes, apparently enhancing pro-oxidative reactions involving ROS. As shown, PMMA polymer caused the destabilization of lysosomal membranes in hemocytes in the experimental mussels and had a lesser effect on the lysosomal membranes of digestive gland and gill cells. Hemocytes are responsible for the immune defense and react instantly to foreign substances and pathogens, in the presence of which the mechanism of their recognition and elimination is triggered in various ways, including the formation of reactive oxygen intermediates and the manifestation of various humoral factors [69]. According to many authors, lysosomal membranes are very sensitive to the oxidative action of ROS, which can be generated by direct and indirect mechanisms involving microplastics [62,63]. For example, when comparing the effects of microparticles ranging in size from 0.5 μm to 3 μm on *M. galloprovincialis* and *Mytilus* spp., the experimental groups showed a significant decrease in phagocytic function with evidence of lysosomal membrane destabilization [67]. The activation of pro-oxidative processes in the cells of experimental mussels after exposure to PMMA microplastics is also evidenced by changes in the low-molecular-weight component of the antioxidant system and the accumulation of the end product of lipid peroxidation, MDA, which is a sensitive indicator of oxidative stress. As far as can be judged from the available literature, these results are consistent with the previously reported pro-oxidative properties of PMMA [40,54,55,60]. In addition to these characteristics, it was shown that microparticles of this polymer significantly enhanced nuclear DNA damage to varying degrees in all studied cells in experimental mollusks. Although genome vulnerability was demonstrated using elevated concentrations of PMMA, this property can persist when exposed to concentrations close to ecologically significant ones, as evidenced by a meta-analysis of literature data on the genotoxicity of various types of microplastics [14]. A distinctive feature of these results is that genome destruction is initiated in the cells of the main tissues of the mussel, which are involved in the interaction with PMMA microparticles. It is also logical to assume that differences in the levels of genome damage may be related to the tissue specificity of DNA repair systems [68]. A popular view in the recent literature is that the genotoxic activity of various types of microplastics can be determined by their ability to induce DNA-damaging ROS [5,10,12,29,70]. Therefore, it is logical to assume that the increase in genomic damage observed in the cells of experimental animals after exposure to PMMA microparticles may also be associated with the pro-oxidant effect of PMMA.

In general, the experimental results presented above indicate the negative consequences that arise when PMMA particles interact with mussels, a typical representative of the marine littoral zone. However, under real environmental conditions, all types of artificial polymers undergo destructive changes to varying degrees as a result of exposure to various environmental factors. Therefore, from an ecotoxicological point of view, the consequences of the interaction of mussels with partially degraded PMMA fragments deserve special attention.

PMMA fragments had relatively prolonged exposure to UV radiation, which is known to initiate photo-oxidative and hydrolytic reactions in various types of synthetic polymers [46]. This approach, which imitates the weathering of polymers under natural conditions, is currently widely used in research to accelerate aging processes and study the ecotoxicological characteristics of polymer degradation [8,9,10,20,22,24,26,47].

To evaluate UV-induced changes in the structure of PMMA, FTIR spectrometry was used, which is currently the main method for detecting chemical changes in the structure of synthetic polymers [71,72,73]. The energy of UV radiation is sufficient to break the C–C and C–H bonds (the bond dissociation energy of C–C and C–H bonds) and activate the oxygen molecules [5], so as a result of UV irradiation, additional oxygen-containing functional groups appear in the structure of the aging polymer. Since such groups are initially present in the structure of PMMA, according to FTIR spectrometry results, UV exposure leads to an enhancement of the 1723 cm^−1^ spectral band corresponding to the carbonyl functional groups. As is known, this broad spectral line represents a carbonyl domain that includes ketone, carboxylic, and aldehyde functional groups [71,72]. The peak at 750 cm^−1^, corresponding to the vibrations of C–H bonds in the methylene groups of the polymer main chain, did not change during UV irradiation and, accordingly, represented the reference spectral band [74]. The OI calculated relative to this peak showed that, as a result of UV irradiation under experimental conditions, the content of carbonyls (C=O bonds) in the composition of PMMA polymer chains increased by 25%, which indicates oxidative degradation of the polymer. Similar changes in the levels of oxygen-containing functional groups are characteristic of both naturally weathered and artificially aged polymers of various types [4,15,75,76]. In addition, it has been established that the oxidative modification of polymer chains significantly affects the chemical (increased negative charge and hydrophilicity) and physical (increased crystallinity, fragmentation, and decreased particle size) characteristics of polymers [8,9,29,67,77,78,79].

In this regard, the increase in the proportion of low-dimensional particles after UV irradiation of PMMA also confirms, to a certain extent, the changes in the physicochemical characteristics of this polymer. It is logical to assume that these changes may be caused by UV-induced fragmentation of high-dimensional polymer particles. However, the influence of physicochemical changes on the polymer surface in the redistribution of PMMA particle size is also possible. Since the hydrocarbon base of PMMA is nonpolar and the hydrophilic ether group is oriented inside the polymer and is inaccessible, the surface of the particles of this polymer is generally highly hydrophobic [74], which contributes to the “sticking” and enlargement of the particles. As a result of UV irradiation, the introduction of oxygen-containing functional groups into the polymer structure, which carry a negative charge, enhances the hydrophilic properties of the polymer surface and prevents the aggregation of microplastic particles.

Experiments showed that the set of changes caused by the artificial photoaging of PMMA had a big impact on its biological activity. At the same time, it should be noted that the nature of the biomarker reaction, reflecting the state of the antiradical link of the antioxidant system and the processes of lipid peroxidation in gill cells and, especially, in the digestive gland, was very weak. This may be due to the activation of the compensatory and detoxification systems, which seriously complicates result interpretation and requires further research. Meanwhile, the behavior of the cytotoxicity and genotoxicity markers indicated an increase in the toxicity of PMMA-UV compared to pristine microplastics. Unlike hemocytes, in the gill and digestive gland cells of experimental mollusks, PMMA-UV microparticles significantly reduced the stability of lysosome membranes compared with pristine PMMA microparticles. Given the functional role of lysosomes, a decrease in the membrane stability of these organelles increases the risk to cell viability and may initiate apoptosis [80].

According to the comet analysis data, PMMA-UV microparticles caused greater genome damage in all studied cells than the pristine microparticles of this polymer. This effect was more pronounced in gill cells, where the average level of nuclear DNA damage induced by the PMMA-UV microparticles was almost twice that caused by the pristine PMMA microparticles. In addition, after exposure of mussels to PMMA-UV, there are cells (about 10%) in the gills with a high level of DNA damage (>30%), which are absent in the gills of mussels exposed to pristine PMMA microparticles. Thus, the enhanced fragmentation of nuclear DNA induced by pristine and photo-oxidized PMMA microparticles demonstrates the genotoxic properties of this polymer, raising serious concerns about unexpected consequences. Obviously, increasing damage to the genome may increase the risk of errors in the biosynthesis of functional biomolecules, which, in turn, may sequentially trigger a cascade of biochemical disturbances leading to serious pathological processes at the upper levels of biological system organization.

Furthermore, within the context of the existing problem, the vulnerability of the somatic cell genome to the effects of artificial polymer microparticles, including PMMA, can be considered a sensitive indicator in determining the minimum effective concentrations for comparison with those actually existing in specific ecosystems.

## 5. Conclusions

In general, the results obtained in this study show that PMMA in the marine environment can be toxic to marine organisms, destabilizing their biochemical processes. This is the first study to show that exposure to UV radiation increases the toxicity of PMMA for *M. trossulus*. This toxicity increase is apparently a consequence of the physicochemical modifications of the PMMA structure caused by irradiation. These results highlight the importance of further studying the effects of the aging process on plastic toxicity, which is needed to assess the future risks to marine ecosystems. Future work in this area should focus on a more detailed study of the relationship between the transformation of plastic particles under the influence of environmental factors and their toxicity to marine organisms. Based on the results, it can be assumed that the biological activity of microparticles of artificial polymers weathered under natural conditions and the risks of negative consequences will be determined not only by their concentration but also by the nature of the oxidative degradation of the polymer structure, which significantly affects the toxicity mechanisms.

## Data Availability

Data is contained within the article.
